# Warneford, Leamington and South Warwickshire Hospital

**Published:** 1911-11-11

**Authors:** 


### Warneford, Leamington and South Warwickshire Hospital.

### Footing the Bill and the Future.

This hospital held an important public meeting at the Town Hall, Leamington, on the 8th inst., at which the President, Lord Algernon Percy, took the chair. He was supported by Lord Leigh, Sir Michael H. Lakin, the Mayor, and most of the members of the Committee and medical staff, including Major H. C. Molyneux and Dr. Thursfield. The hall was well filled, and the proceedings most interesting. Lord Algernon Percy made a most practical speech and justly commended the history of the hospital prepared by the House Governor and Secretary, Mr. Fred Smith. He showed that during the last twenty years the hospital has been much extended and practically rebuilt at a cost of £35,000, that £24,000 has been added to the investments, and £4,500 of capital, or an average of only £225 per annum, has been taken from capital account to meet deficiencies. The hospital is now thoroughly well found and administered, and Lord Percy asked for and should speedily receive £3,000 in cash and £1,000 in increased revenue.

